# Effect of smoking cessation on sexual functions in men aged 30 to 60 years

**DOI:** 10.1590/S1677-5538.IBJU.2019.0541

**Published:** 2020-03-25

**Authors:** Mehmet Oguz Sahin, Volkan Sen, Gazi Gunduz, Oktay Ucer

**Affiliations:** 1 Department of Urology Manisa State Hospital Manisa Turkey Department of Urology, Manisa State Hospital, Manisa, Turkey;; 2 Department of Chest Diseases Manisa State Hospital Manisa Turkey Department of Chest Diseases, Manisa State Hospital, Manisa, Turkey;; 3 Department of Urology Manisa Celal Bayar University School of Medicine Manisa Turkey Department of Urology, Manisa Celal Bayar University School of Medicine, Manisa, Turkey

**Keywords:** Erectile Dysfunction, Sexual Development, Smoking

## Abstract

**Purpose:**

We aimed to evaluate the effects of smoking cessation on the sexual functions in men aged 30 to 60 years.

**Materials and Methods:**

Male patients aged 30 to 60 years that presented to the smoking cessation polyclinic between July 2017 and December 2018 were prospectively included in the study. The amount of exposure to tobacco was evaluated in pack-year. The patients filled the International Index of Erectile Function (IIEF) form before the cessation and six months after cessation of smoking. Patients were subgrouped according to age, education level and packs/year of smoking and this groups were compared in terms of IIEF total and all of the IIEF domains.

**Results:**

The evaluations performed by grouping the patients according to age (30-39, 40-49 and 50-60 years) and education level (primary-middle school and high school-university) revealed that the total IIEF scores obtained after smoking cessation were significantly higher compared to the baseline scores in all groups (p=0.007 for the 30-39 years group and p <0.001 for the remaining groups). According to grouping by exposure to smoking (≤25, 26-50, 51-75, 76-100 and 101≥ packs/year), the total IIEF scores significantly increased after smoking cessation in all groups except 101≥ packs/year (p=0.051 for the 101≥ group and p <0.001 for the remaining groups).

**Conclusions:**

Erectile function is directly proportional to the degree of exposure to smoking, and quitting smoking improves male sexual function in all age groups between 30-60 years of age regardless of pack-year and education level.

## INTRODUCTION

Smoking, a widely present addiction around the World, can cause important health problems. It is reported that tobacco products contain around 4.000 chemical compounds, of which at least 60 are toxic ( 1 ). Many studies have shown the relationship between smoking and hypertension, acute coronary syndrome, angina, atherosclerosis, cerebrovascular diseases, and sudden death ( 2 ). Although the mechanism of this relationship has not yet been fully elucidated, it has been reported to lead to atherosclerosis as a result of vasomotor dysfunction, inflammation, and modification of lipids ( 3 ).

Erectile dysfunction (ED) is defined as the inability to achieve or maintain penile erection of adequate quality to achieve satisfactory sexual intercourse. ED is not a direct threat to life, but it should also not be seen as a benign disorder because it is increasingly associated with cardiovascular diseases, such as ischemic cerebrovascular events, angina pectoris, myocardial acute insufficiency, and sudden death. Some authors have suggested that ED is a sentinel event and an early marker of cardiovascular diseases ( 4 ). According to the Massachusetts Male Aging Study (MMAS), 52% of men aged 40 to 70 years present varying degrees of erectile dysfunction ( 5 ). Endothelial dysfunction and microvascular damage play a role in the pathogenesis of ED. Among the main risk factors for this condition are high systolic blood pressure, diabetes, obesity, smoking, and dyslipidemia. It is known that the significant risk factors associated with ED are also frequently seen in smokers ( 6 ). Reducing smoking, engaging in regular exercise, adopting a healthy diet, losing excess weight, controlling diabetes, and making positive lifestyle changes have proven to reduce the risk of ED and metabolic syndrome ( 7 ).

There are several hypotheses on the physiopathological effects of long-term smoking on sexual dysfunction. Smooth muscle relaxation due to sexual arousal is a complex neurovascular event, in which arterial access to the genital area is provided and vasocongestion is facilitated ( 8 ). Nitric oxide (NO) produced in the genital endothelial cells has been defined as the main neurotransmitter mediating vascular events ( 9 , 10 ). It has also been shown that smoking is associated with decreased NO in the veins ( 11 ). In light of these findings, researchers have suggested that free radicals and other compounds present in tobacco products may reduce the synthesis of NO either directly or indirectly by targeting precursors, which leads to a decrease in genital vaso-occlusion ( 12 - 14 ).

In this study, we aimed to evaluate the effects of smoking cessation on the sexual functions of men aged 30 to 60 years.

## MATERIALS AND METHODS

Following the approval of the ethics committee, male patients aged 30 to 60 years that presented to the smoking cessation polyclinic between July 2017 and December 2018 were prospectively included in the study. The inclusion criteria were: having no psychiatric disease, not using alcohol or drugs, having no systemic disease, having no history of surgery, having a body mass index (BMI) of 20 to 25kg/m2, not using any tobacco product after cessation of smoking, not being a passive smoker, not having received any medical or surgical treatment for ED, and having a regular sexual partner. The amount of exposure to tobacco was evaluated in pack-year. Drugs for smoking cessation were given to the patients according to the avalibility of drugs in the hospital. The majority of patients (172/181, 95%) used Varenicline 1mg tablets 2x1/day and a small number of patients (9/181, 5%) received Bupropion HCL 150mg tablets 2x1/day for three months. The patients were asked to complete the International Index of Erectile Function (IIEF) form before and six months after cessation of smoking. In the erectile function (EF) domain of the IIEF questionnaire (items 1, 2, 3, 4, 5 and 15, range 0-5, max score 30), a score lower than 10 indicates severe ED, 11-16 moderate ED, 17-25 mild ED, and 26-30 normal EF. In the evaluation of IIEF-EF stage improvement, transitions from severe to moderate ED, from moderate to mild ED, or from mild ED to normal EF groups were accepted as improvement (+) in EF. The remaining domains of IIEF are intercourse satisfaction containing items 6 to 8 (range 0-5, max score 15), orgasmic function with items 9 and 10 (range 0-5, max score 10), sexual desire with items 11 and 12 (range 0-5, max score 10), and overall satisfaction with items 13 and 14 (range 0-5, max score 10). Patients were subgrouped according to age, education level and packs/year of smoking and these groups were compared in terms of IIEF total and all of the IIEF domains.

The analyses of data were performed with the Statistical Package for the Social Sciences software for Windows (SPSS, Inc., Chicago IL) version 22, and the data were presented as mean±standard deviation and numbers (n) and percentages (%). Student’s paired t-test was used for the comparison of the domain scores of the IIEF questionnaire before and after smoking cessation and one-way ANOVA test to evaluate the association between smoking exposure and ED severity. P values of <0.05 were considered as statistically significant.

## RESULTS

A total of 202 patients were evaluated, 21 of them restarted to smoke and were excluded from the study, and finally 181 patients were included in the study. The mean age of patients was 47.7±9.6 (min 30-max 60) years, and the mean pack-year was 46.1±32.2 (min 5-max 160). The total IIEF score was 54.8±16.7 (min 9-max 75) before smoking cessation and 60.4±15.3 (min 15-max 75) after smoking cessation.

The evaluations performed by grouping the patients according to age (30-39, 40-49 and 50-60 years) and education level (primary-middle school and high school-university) revealed that the total IIEF scores obtained after smoking cessation were significantly higher compared to the baseline scores in all groups (p=0.007 for the 30-39 years group and p ˂0.001 for the remaining groups) ( Table-1 ). According to grouping by exposure to smoking (≤25, 26-50, 51-75, 76-100 and 101≥ packs/year), total IIEF scores were significantly increased after smoking cessation in all groups except 101≥ packs/year (p=0.051 for the 101≥ group and p ˂0.001 for the remaining groups) ( Table-1 ). Stage improvement was observed in 25.4% of the patients, but no statistically significant difference was found between the age groups, pack-year groups, or education level groups (p=0.124, p=0.052 and p=0.475, respectively) ( Table-1 ).

**Table 1:** Comparison of the total IIEF scores and stage improvement status before and after smoking cessation in age, pack-year and education level groups.

	Smoking (+)	Smoking (-)	P	Stage	Stage	p

	Total IIEF score (mean ± SD)	Total IIEF score (mean ± SD)		improvement (-) 135 (74.6%)	improvement (+) 46 (25.4%)	
**Age groups (years)**						0.124
1) 30-39 (n=38)	27.4 ± 4.4	28.9 ± 2.6	0.007	33 (86.8%)	5 (13.2%)	
2) 40-49 (n=47)	23.8 ± 4.1	26.2 ± 3.5	<0.001	35 (74.5%)	12 (25.5%)	
3) 50-60 (n=96)	18.3 ± 7.7	21.4 ± 7.4	<0.001	67 (69.8%)	29 (30.2%)	
**Pack-year groups**						0.052
1) 25≤ (n=63)	23.3 ± 7.9	25.6 ± 6.8	<0.001	44 (69.8%)	19 (30.2%)	
2) 26-50 (n=56)	22.7 ± 5.2	26.1 ± 4.1	<0.001	44 (78.6%)	12 (21.4%)	
3) 51-75 (n=27)	19.0 ± 8.4	20.9 ± 6.8	<0.001	22 (81.5%)	5 (18.5%)	
4) 76-100 (n=25)	18.3 ± 7.6	20.8 ± 8.0	<0.001	21 (84.0%)	4 (16.0%)	
5) 101≥ (n=10)	20.2 ± 6.5	22.6 ± 6.3	0.051	4 (40.0%)	6 (60.0%)	
**Education level groups**						0.475
1) Primary - middle school (n=79)	22.5 ± 7.5	24.9 ± 7.0	<0.001	61 (71.2%)	18 (28.8%)	
2) High school - university (n=102)	21.0 ± 7.2	23.7 ± 6.2	<0.001	74 (72.5%)	28 (27.5%)	

In a separate comparison undertaken according to the IIEF domain scores, it was found that all domain scores significantly increased after smoking cessation ( Table-2 ).

**Table 2:** IIEF = International Index of Erectile Function - Comparison of the IIEF domain scores before and after smoking cessation.

IIEF domains (item number)	Smoking (+)	Smoking (-)	P

	IIEF domain score (mean±SD)	IIEF domain score (mean±SD)	
EF (1,2,3,4,5,15)	21.6 ± 7.3	24.2 ± 6.6	<0.001
Intercourse satisfaction (6,7,8)	10.5 ± 3.5	11.7 ± 3.2	<0.001
Orgasmic function (9,10)	8.6 ± 2.4	8.9 ± 2.1	<0.001
Sexual desire (11,12)	6.8 ± 2.0	7.7 ± 1.9	<0.001
Overall satisfaction (13,14)	7.2 ± 2.5	8.0 ± 2.0	<0.001

**IIEF** = International Index of Erectile Function, **EF** = Erectile Function

The IIEF-EF scores also significantly increased in the severe, moderate and mild ED groups, but not in the normal-EF group after smoking cessation ( Table-3 ).

**Table 3:** Comparison of the IIEF-EF domain scores of the IIEF-EF categories before and after smoking cessation.

IIEF-EF categories (baseline evaluation)	Smoking (+)	Smoking (-)	p

	IIEF-EF domain score (mean±SD)	IIEF-EF domain score (mean±SD)	
1) Severe ED (score ≤10) (n=14)	5.2 ± 2.4	8.2 ± 3.9	0.009
2) Moderate ED (score 11-16) (n=19)	12.5 ± 1.0	16.7 ± 3.8	<0.001
3) Mild ED (score 17-25) (n=93)	21.3 ± 2.8	24.9 ± 3.2	<0.001
4) Normal EF (score 26-30) (n=55)	29.5 ± 1.2	29.7 ± 0.8	0.061

**IIEF-EF** = International Index of Erectile Function-Erectile Function, **ED** = Erectile dysfunction

When severe ED, moderate ED, mild ED and normal EF groups determined according to the IIEF-EF domain score were compared in terms of the mean pack-year, it was seen that EF deteriorated with increasing exposure to smoking (p *˂* 0.001) ( Table-4 ).

**Table 4:** Comparison of the IIEF categories according to exposure to smoking.

IIEF-EF categories (baseline evaluation)	Exposure to smoking (packs/year) (mean±SD)	P
1) Severe ED (score ≤10) (n=14)	57.1 ± 39.6	< 0.001
2) Moderate ED (score 11-16) (n=19)	53.3 ± 23.8	
3) Mild ED (score 17-25) (n=93)	51.5 ± 35.4	
4) Normal EF (score 26-30) (n=55)	31.6 ± 20.6	

**IIEF-EF =** International Index of Erectile Function-Erectile Function, **ED** = Erectile dysfunction

## DISCUSSION

Penile erection is largely caused by the presence of sufficient blood flow into the erectile tissue, simultaneous arterial endothelium-dependent dilatation, and sinusoidal endothelium-dependent corporal smooth muscle relaxation ( 15 ). Free radicals, aromatic compounds and superoxide anions in the smoke of tobacco products can disrupt dilation by impairing NO synthesis and degradation in the penile artery and arterioles ( 16 ). In addition, smoking is an independent risk factor for atherosclerosis in internal, pudental and common penile arteries ( 17 ). Considering these mechanisms, the development of ED is an expected outcome in smokers. Furthermore, it is suggested that the risk of ED increases with the elevated amount of exposure to cigarette toxins, smoking accompanied by aging, and cavernosal arterial occlusive conditions, such as hypertension and diabetes mellitus ( 5 , 17 ).

Nicotine replacement therapy and non-nicotine drugs are the most commonly used pharmacological treatments in tackling smoking addictions. Bupropion is a well-tolerated medication used in smoking cessation to reduce withdrawal symptoms during treatment and weight gain after quitting smoking ( 18 , 19 ). Varenicline also has nicotinic agonist effects that stimulate α4 β2 receptors and provide dopamine release from the nucleus accumbens, which is followed by the antagonistic effect, meaning that there is no increase in dopamine release even if the person inhales nicotine when using varenicline. Through these agonist and antagonist functions, varenicline decreases nicotine dependence and prevents the occurrence of withdrawal symptoms ( 20 ).

Mannino et al. reported that the incidence of ED increased in smokers and decreased after smoking cessation in their study conducted with 4.500 Vietnam War veterans ( 21 ). Guay et al. found that in patients who previously smoked more than 30 packs/year, there was rapid improvement in penile integrity and rigidity one month after smoking cessation. The authors noted that according to the study data, this improvement was more significant in the younger age group and in the absence of additional diseases that might pose risk for ED ( 22 ). In contrast, in our study, we found that improvement was more significant in the older age group. This may be due to the absence of additional systemic diseases and the maximum age of our sample being 60 years.

Pourmand et al. investigated the effects of smoking cessation and continuation of the non-smoker status in patients with ED. They found that the severity of ED was significantly related to the level of exposure to smoking. After one year of follow-up, the authors detected improvement in EF in ≥25% of ex-smokers but in none of the persistent smokers. Furthermore, 2.5% of the ex-smokers and 6.8% of persistent smokers had deterioration in the ED status. Better EF was observed in the follow-up of ex-smokers. It was also reported that among those who stopped smoking, older cases had the least improvement in EF ( 23 ). In the current study, we only included patients that stopped smoking and did not start it again. Fifty-five of the patients that were followed up (30.4%) consisted of ex-smokers with normal EF. We found a direct correlation between exposure to smoking (pack/year) and the negative effect of smoking on EF. In terms of smoking categories according to package/years, the total IIEF scores positively increased in all groups after smoking cessation. Furthermore, this increase was not statistically significant only in the ≥101 packs/year group (excessive exposure to smoking). In our study, we found no positive effect of stopping smoking on the EF of patients with a normal IIEF-EF score (26 to 30) before smoking cessation. However, in all categories of IIEF-EF, we detected positive improvement after smoking cessation. In addition, we detected 25.4% stage improvement similar to the result reported by Pourmand et al. In contrast, in the current study, we did not observe any ED at the end of six months. Moreover, contrary to Pourmand et al., the greatest stage improvement (30.2%) occurred in the elderly group of our study (50-60 years). We also found that stage improvement was not significantly correlated with age, exposure to smoking, and education level. As an additional finding of our study, we found improvement not only in the IIEF-EF domain but also in the evaluation of intercourse satisfaction, orgasmic function, sexual desire, and overall satisfaction after smoking cessation. This may be due to not only improved EF, but also changes in serum testosterone levels with the discontinuation of smoking, although there are conflicting reports in the literature ( 24 - 27 ).

The main limitation of this study was that early (e.g., first-month) and late outcomes after smoking cessation were not evaluated. The second limitation of study was the restrictive age group of patients; the results can not be generalized to older patients. However, the advantage of the study was the presence of a patient group independent of other factors that may affect EF.

## CONCLUSIONS

In conclusion, EF is directly proportional to the degree of exposure to smoking, and stopping smoking improves male sexual function in all age groups between 30-60 years of age regardless of number of packs-year and education level.
